# Variation of Growth-to-Ripening Time Interval Induced by Abscisic Acid and Synthetic Auxin affecting Transcriptome and Flavor Compounds in Cabernet Sauvignon Grape Berry

**DOI:** 10.3390/plants9050630

**Published:** 2020-05-14

**Authors:** Lei He, Zhi-Yuan Ren, Yu Wang, Ya-Qun Fu, Yue Li, Nan Meng, Qiu-Hong Pan

**Affiliations:** 1 Center for Viticulture & Enology, College of Food Science and Nutritional Engineering, China Agricultural University, Beijing 100083, China; helei@cau.edu.cn (L.H.); Rzy@cau.edu.cn (Z.-Y.R.); wangyu_0919@cau.edu.cn (Y.W.); fuyaqun@cau.edu.cn (Y.-Q.F.); sq18813017889@cau.edu.cn (Y.L.); mn@cau.edu.cn (N.M.); 2 Key Laboratory of Viticulture and Enology, Ministry of Agriculture and Rural Affairs, Beijing 100083, China; 3 College of Food Science and Engineering, Shanxi Agriculture University, Jinzhong 030801, China

**Keywords:** grape berry, ABA, NAA, anthocyanin, volatile compound

## Abstract

Abscisic acid (ABA) and auxin are important hormones controlling the ripening progression of grape berry, and both the initiation and duration of ripening can dramatically affect the berry quality. However, the responses of flavor compounds to the hormones are inadequately understood. In this study, ABA and synthetic auxin α-naphthaleneacetic acid (NAA) were sprayed on Cabernet Sauvignon berries before véraison, and comparative transcriptomic and metabolic analysis were conducted to investigate the influence on berry quality-related metabolites. The 1000 mg/L ABA (ABA1000) and 200 mg/L NAA (NAA200) treated grapes exhibited shorter and longer phenological intervals compared to the control, respectively. The transcriptomic comparison between pre-véraison and véraison revealed that the varied ripening initiation and duration significantly affected the expression of genes related to specific metabolism, particularly in the biosynthetic metabolism of anthocyanin and volatile compounds. The up-regulated *VviF3’H* in both ABA1000-treated and NAA200-treated berries increased the proportion of 3′-substituted anthocyanins, and the 3′5′-substituted anthocyanins were largely reduced in the NAA200-treated berries. Concurrently, *VviCCD4a* and *VviCCD4b* were up-regulated, and the norisoprenoids were correspondingly elevated in the NAA200-treated berries. These data suggest that ABA and NAA applications may be useful in controlling the ripening and improving the flavor of the grape berry.

## 1. Introduction

Timing and rate of ripening are key factors in grape berries for winemaking. The composition and concentration of flavor compounds in both grapes and wine are closely associated with the ripening process and harvest maturity [1,2,3]. Immature grape berries generally contain high concentrations of organic acids, volatile C6 compounds, and methoxypyrazines, which are undesirable [4]. Furthermore, the ripening rate appears to affect the accumulation of flavor compounds. In warm regions with the rapid accumulation of soluble solids, harvested grape berries fail to develop adequate and desirable flavors, even with the optimum sugar/acidity ratios [5,6]. On the contrary, grape berries in cooler regions need longer times to achieve industrial maturity, possibly due to the lack of adequate sunlight [7]. The ripening process can thus be influenced by many factors such as variety, light, water status, temperature, and hormones [8]. However, the knowledge about the relationship between hormones and flavor compounds, especially volatile aroma compounds, is limited.

Endogenous abscisic acid (ABA) in grape berries begins to increase at around véraison, followed by a decrease when full ripening approaches [9,10,11]. In contrast, the concentration peak of endogenous auxin appears during the flowering period, and then dramatically declines at véraison [12]. Endogenous ABA and auxins act as the inducer and repressor of ripening inception, respectively. The application of ABA at pre-véraison can advance the accumulation of sugar and the berry ripening process [11], whereas spraying with auxin-like compounds (such as α-naphthaleneacetic acid; NAA) has the opposite effect [9,13,14]. The integrated analyses of the transcriptome and metabolome indicate that the dramatic changes in the transcription rate of a large number of genes and the production of flavor metabolites do occur in the grape berries treated with ABA or auxin-like substances [15,16]. Exogenous ABA can stimulate the biosynthesis of anthocyanins, elevating their concentration [15,17], whereas NAA treatments cause the opposite effects [13]. However, in published research, the data comparison between hormone treatments and control groups, to our knowledge, is based on a specific date after treatment at which the berries actually possess different soluble solid contents. For example, Koyama et al. found that ABA treatment stimulates the rapid production of anthocyanins and flavonols in comparison with controls at 14, 28, and 37 days after véraison [15]. Previous studies of exogenous ABA have concentrated on flavonoids and their biosynthetic-related genes. Few reports have shown that there is a significant difference between the volatile compounds in the wines produced from NAA-treated and control berries [13,18]. Volatile compounds, such as terpenes, are major contributors to the floral/fruity attributes of grape berries, and their levels have been considered indicators for distinguishing muscat, non-muscat, and neutral varieties [19]. Norisoprenoids are also important volatile compounds with very low sensory thresholds, which allows their flavor notes to be easily incorporated into the overall aroma of grapes [20,21]. Knowledge of the responses of these volatile compounds to exogenous ABA or NAA can help us understand berry ripening and contribute to grape and wine quality improvement.

Véraison, the shift from berry growth to berry ripening, is of considerable scientific interest as it is a period of dramatic physical and chemical changes. These changes are triggered by a complex interplay between hormones and accompanied by the regulation and accumulation of flavor compounds [22,23,24]. The objective of the study was to evaluate the responses of the transcriptome and flavor metabolome to ABA and NAA during the transition from berry growth to ripening. The results indicate that ABA can promote the accumulation of 3′-substituted anthocyanins, and NAA treatment can benefit the biosynthesis and accumulation of norisoprenoids. This finding provides a strategy for volatile aroma quality improvement for wine grape production.

## 2. Results

### 2.1. Variation in Timing and Duration of Ripening by ABA and NAA Treatments

**Figure 1:**
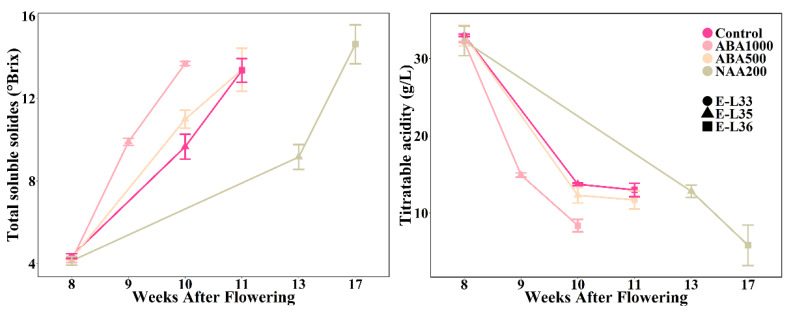
Changes of total soluble solids and titratable acidity in the control, ABA1000-treated, ABA500-treated and NAA200-treated grape berries.

Spraying ABA1000 on berry clusters resulted in a sharp increase in the total soluble solid (TSS) content and a synchronous decline of the titratable acidity (Figure 1). Ripening of the ABA1000-treated berries started nine weeks after flowering, about one week ahead of the controls. In contrast, NAA200 caused a slower accumulation of TSS and a decline in titratable acidity (TA). Correspondingly, the ripening initiation (E-L 35) of NAA-treated grape berries was delayed by about three weeks, and the interval between E-L 35 and E-L 36 stages was prolonged three weeks. Surprisingly, the ABA500-treated berries exhibited similar developmental progression with the control berries (Figure 1). The application of NAA led to a slightly lower TA at the E-L 36 stage in comparison with control berries.

### 2.2. Transcriptomic Analysis of Gene Expression Profiling

**Figure 2:**
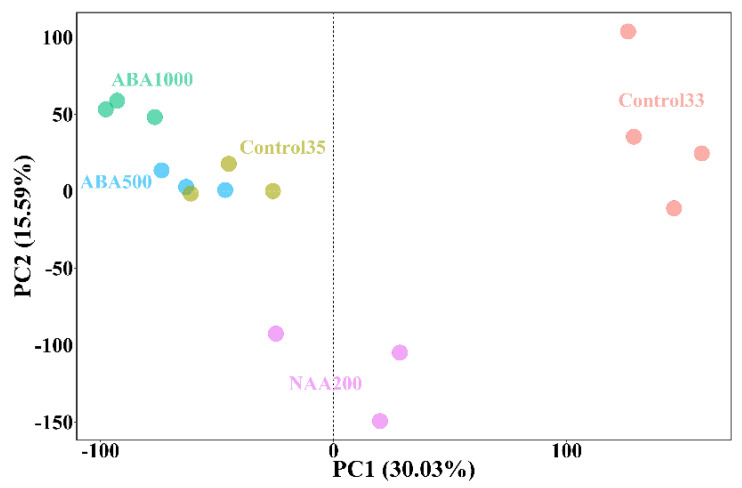
Principal component analysis of the ABA-treated, NAA-treated, and control groups. Variables are the transcript abundance of 27,267 genes. Each circle represents a biological replicate. Control33 and Control35 represent the samples without treatments that were collected at the E-L 33 and 35 stages, respectively. ABA1000, ABA500, and NAA200 represent the samples treated by 1000 mg/L ABA, 500 mg/L, 200 mg/L NAA, respectively, that were collected at the E-L 35 stage.

A total of 27,267 genes were annotated from the RNA-seq reads across all the samples. Four biological replicates were performed for the Control33, and three biological replicates were conducted for each of the other four groups for E-L 35 stage. Principal component analysis (PCA) was carried out using reads per kilobases per million reads (RPKM) of all detected genes to the genome-scale differences. The first two principal components (PCs) explained 45.62% of the total variance. The Control33 was separated from other groups by PC1 and PC2, and the NAA200 at the E-L 35 stage was also scattered by PC2 with a high negative score (Figure 2). This distribution pattern illustrated that a large alteration of transcriptomes occurred between the E-L 33 and E-L 35 stages. More importantly, at véraison, the gene expression in the NAA-treated grape berries differed from those in the ABA-treated and control berries.

**Figure 3:**
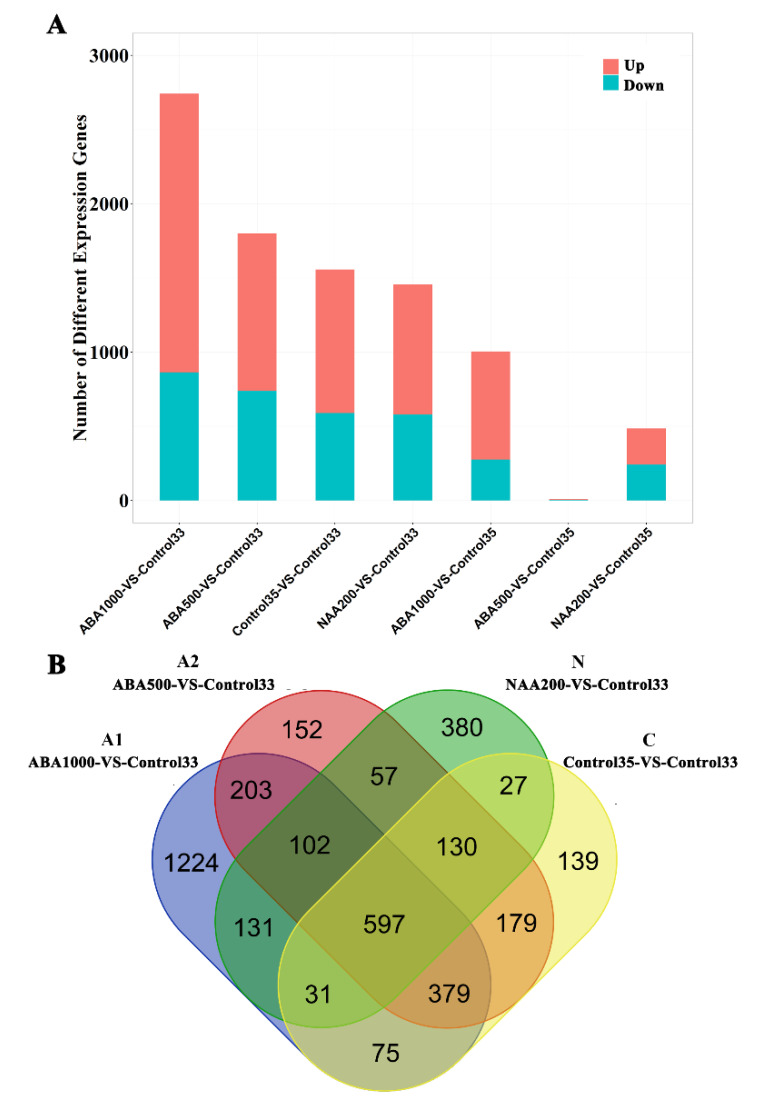
(**A**) Differentially expressed genes (DEGs) between the different samples. Former four bars represent the number of DEGs in the E-L 35 stage (ripening initiation) relative to Control33; last three bars represent the number of DEGs in the E-L 35 stage compared to the samples of Control35. Red and cyan bars represent the number of up-regulated and down-regulated genes, respectively. (**B**) Venn diagram summary of the four DEG sets: A1, ABA1000 vs. Control33; A2, ABA500 vs. Control33; C, Control35 vs. Control33; N, NAA200 vs. Control33.

To illustrate the mechanisms underlying different ripening rates, four sets of differentially expressed genes (DEGs) were identified, including A1 (ABA1000 vs. Control33), A2 (ABA500 vs. Control33), C (Control35 vs. Control33), and N (NAA200 vs. Control33). There were 1,878, 1,060, 967 and 876 up-regulated genes and 864, 739, 590, and 579 down-regulated genes in the ABA1000, ABA500, Control35, and NAA200 berries, respectively (Figure 3A). The greatest number of DEGs was between ABA1000 and Control33, while the smallest number was between NAA200 and Control33 (Figure 3A). The comparison between Control35 and the three treated groups at E-L 35 showed that there were 728 and 243 genes up-regulated as well as 276 and 243 genes down-regulated with the ABA1000 and NAA200 treatments (Figure 3A). However, only five genes were up-regulated, and four genes were down-regulated in the ABA500-treatment, relative to Control35. 

### 2.3. Genes Associated with Growth-To-Ripening Transition and Ripening Initiation

**Figure 4:**
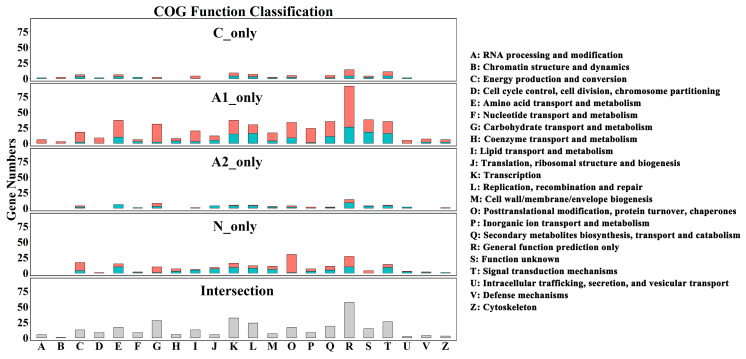
Functional classification of differentially expressed genes (DEGs) in C_only, A1_only, A2_only, N_only, and intersections of DEG sets of A1, A2, C, and N based on clusters of orthologous groups of proteins (COG) annotation. C_only: DEGs only found in the Control35 vs. Control33; A1_only: DEGs specifically present in ABA1000 vs. Control33; A2_only: DEGs specifically present in ABA500 vs. Control33; N1_only: DEGs specifically present in NAA200 vs. Control33. Red and cyan bars, respectively, represent the number of up-regulated and down-regulated genes in the treated grape berries of the E-L 35 stage (ripening initiation), compared to Control35.

There were 597 common DEGs across the four sets of A1, A2, C, and N (Figure 3B). There were 380, 1,224, 152, and 139 genes that were only found in the sets of C, A1, A2, and N, respectively, and were named as C_only, A1_only, A2_only, and N_only. From the annotation of the clusters of orthologous groups of proteins (COG) database, the 597 commonly-expressed genes mainly belonged to the functional categories of “transcription”, “replication, recombination, and repair”, “carbohydrate transport and metabolism”, “amino acid transport and metabolism”, “secondary metabolites biosynthesis, transport and catabolism”, and “signal transduction mechanisms” (Figure 4), suggesting that these processes were associated with the activation of grape berry ripening. Interestingly, the DEGs of A1_only exhibited a similar functional pattern with those commonly expressed genes, and most showed high expression levels. Whereas the DEGs of the N _only were related to the categories of “energy production and conversion” and “post-translational modification, protein turnover, chaperones”. Small numbers of C_only and A2_only DEGs were annotated in various COG categories (Figure 4). 

**Figure 5:**
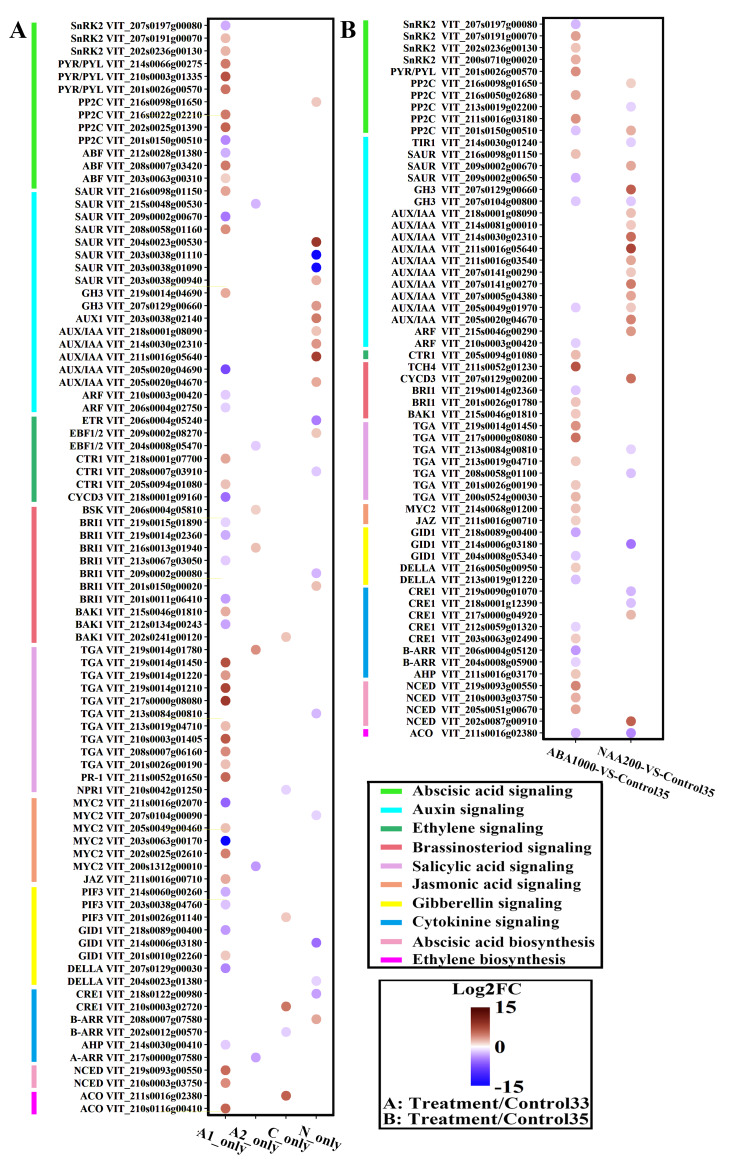
Expression of the genes responsible for hormone biosynthesis and signaling. (**A**): Differentially expressed genes (DEGs) only exist in sets of A1, A2, C, and N. (**B**): DEGs in sets of ABA1000 vs. Control35 and NAA200 vs. Control35. The colors of the circles represent the intensity of the expression fold changes (log2). A1_only: DEGs specifically present in the comparison of ABA1000 vs. Control33; A2_only: DEGs specifically present in the comparison of ABA500 vs. Control33; C_only: DEGs specifically present in the comparison of Control35 vs. Control33; N_only: DEGs specifically present in the comparison of NAA200 vs. Control33.

Berry softening, sugar accumulation, catabolism of acid, and increase in ABA content are important physiological changes during the inception of ripening. In the A1_only set, several genes encoding pectin esterase (PE) and polygalacturonase (PG), were significantly up-regulated (Table S1). In addition, beta-fructofuranosidase genes, reported to have a capacity for hydrolyzing sucrose [25], were significantly up-regulated in ABA1000 grape berries. The ABA1000 grapes also exhibited an up-regulation of genes that were reported to encode for sugar transporters. While in the N_only, no known genes relating to berry softening and sugar transporters were found (Table S1). In the present study, one gene (VIT_203s0088g01190) in N_only, encoding for malate dehydrogenase (MDH), was up-regulated at the transcriptional level and may contribute to the significant decline of acidity in NAA200-treated berries at E-L 35 (Figure 1). Moreover, further comparisons of ABA1000 vs. Control35 and NAA200 vs. Control35 reflect the transcriptome differences at véraison induced by ABA1000 and NAA200. The ABA1000-treated berries presented higher expression levels of some *VviPE*, *VviPL*, and beta-fructofuranosidase genes and VIT_218s0076g00250, encoding a sucrose transporter (SUC). In contrast, the expression of 3 *VviPE* (VIT_209s0002g00330, VIT_215s0048g00510, and VIT_217s0000g05960), 1 *VviPG* (VIT_208s0007g08330), and one gene encoding sucrose synthase (SUS) were down-regulated in the NAA200-treated berries, while 3 *VviMDH* exhibited higher expression levels. It was noticed that in the set of A1_only, most of the genes coding for these proteins showed significantly increased expression levels (Figure 5A). By contrast, the gene for brassinosteroid-insensitive1 (BRI1) showed down-regulated expression. In the set of N_only, the genes encoding one auxin influx carrier protein (AUX1) and four auxin-responsive proteins (AUX/IAA) showed increased levels, and a few genes were found to be involved in hormone synthesis and signaling in A2_only and C_only. Hormonal regulation can also be found at the E-L 35 stage (Figure 5B). Besides the genes relating to the synthesis and signaling of ABA and SA, one gene of xyloglucan—xyloglucosyl transferase (TCH4)—also exhibited a very high transcript level in the ABA1000 grapes of E-L 35. By contrast, the genes relating to auxin signaling were markedly up-regulated by NAA200.

The present study indicates that even though grape berries all reached the véraison stage with similar physiological phenotypes, the genes associated with each’s ripening-related metabolism exhibited large differences in expression abundance between the treated and control groups.

### 2.4. Identification of Development-Specific and Treatment-Specific Metabolites

**Figure 6:**
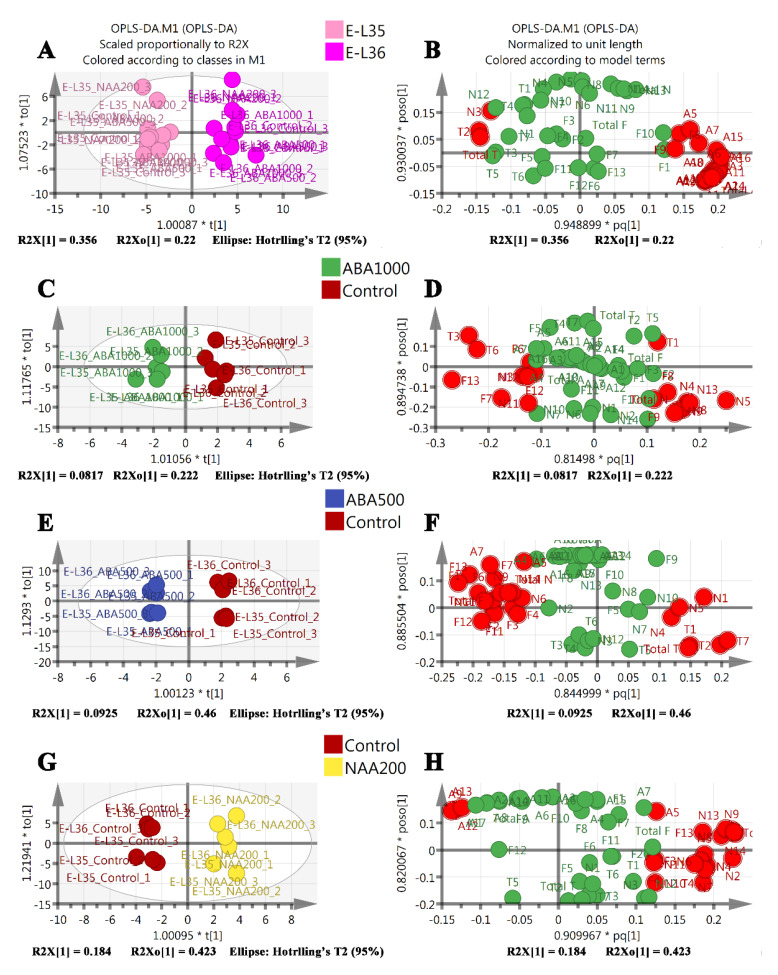
Supervised (developmental stage and treatments) orthogonal partial least squares-discriminant analysis (OPLS) of all metabolites from the stages of E-L 35 (ripening initiation) and E-L 36 (berries with intermediate Brix values). (**A**) Score plot of the respective samples, developmental stages are highlighted with different colors. (**C**) Score plot for the respective samples, ABA1000, and control group are highlighted with different colors. (**E**) Score plot for the respective samples, ABA500 and control group are highlighted with different colors. (**G**) Score plot for the respective samples, NAA200 and control group are highlighted with different colors. (**B**, **D**, **F**, **H**) Loading plot for the measured variables in green. Red spots represent the variables significantly contributing to the models with variable importance plot (VIP) values larger than 1.

Orthogonal partial least squares-discriminant analysis (OPLS-DA) models were used to identify the optimal model variables for the development stage discriminant and treatment discriminant. Variable importance for the projection (VIP) value ranks the overall contribution of each variable to the OPLS-DA model [26]. VIP values larger than 1 indicate “important” X-variables. According to the VIP values of the variables (Table S2)—the metabolites that had the greatest contribution to the model of discriminant development were the anthocyanins (Figure 6A,B); for the model of ABA1000-specific discriminant they were hexanoic acid, vitispirane B, and linalool (Figure 6C,D); for the model of ABA500-specific discriminant they were hexanoic acid, alpha-terpineol, and hexanal (Figure 5E,F); for the model of the NAA200-specific discriminant they were (*Z*)-*β*-damascenone, total norisoprenpoids, TCH, and (*E*)-*β*-damascenone (Figure 6G,H). A list of compounds corresponding to the symbols in the loading plots is available in Table S3.

**Figure 7:**
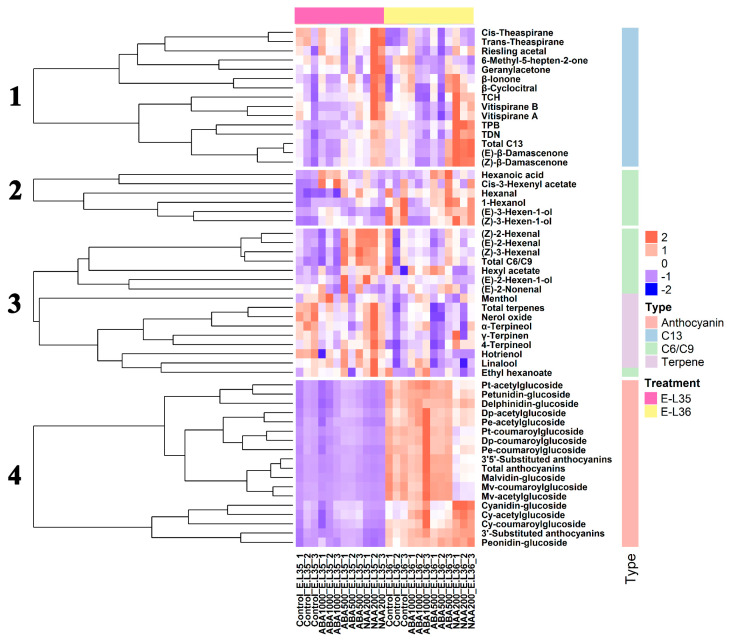
Heatmap analysis and hierarchical clustering of anthocyanins, terpenes, norisoprenoids, and C6/C9 compounds.

Hierarchical cluster analysis was performed to identify clusters with similar trends among the analyzed metabolites (Figure 7). Similar to the results of OPLS-DA, all norisoprenoids exhibited a predominant treatment effect and were obviously increased in NAA200-treated berries (cluster 1). Terpenes and volatile C6/C9 compounds showed both developmental and treatment effects (cluster 2 and cluster 3); for example, the concentrations of 1-hexanol and (*E*)-3-hexen-1-ol in the berries of treatments and controls were increased from E-L 35 to E-L 36, and both of them showed lower levels in ABA1000-treated berries compared to the control at the E-L 36 stage. Similarly, terpenes such as nerol oxide, hotrienol, and linalool decreased during the ripening, and these terpenes were reduced by ABA1000 at the E-L 35 stage. All detected anthocyanins in cluster 4 were developmentally controlled.

**Figure 8:**
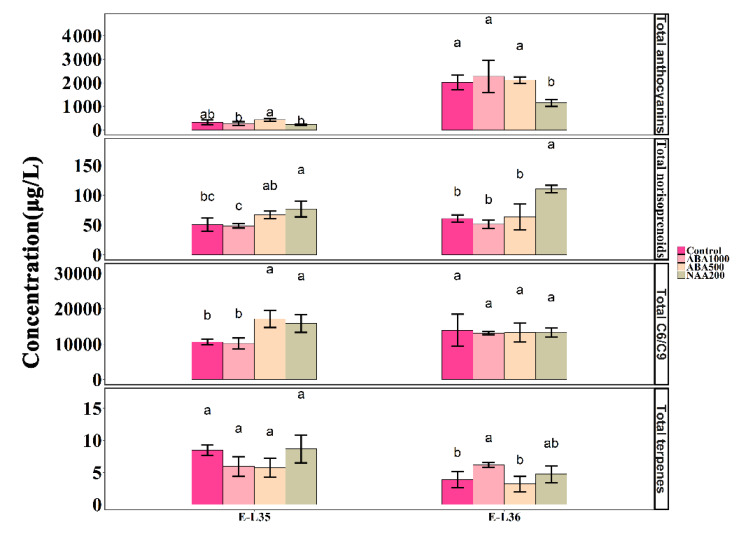
Changes in the concentrations of total anthocyanins, total norisoprenoids, total C6/C9 compounds, and total terpenes in the different treatments. Different letters indicate significant differences (*p* = 0.05).

**Figure 9:**
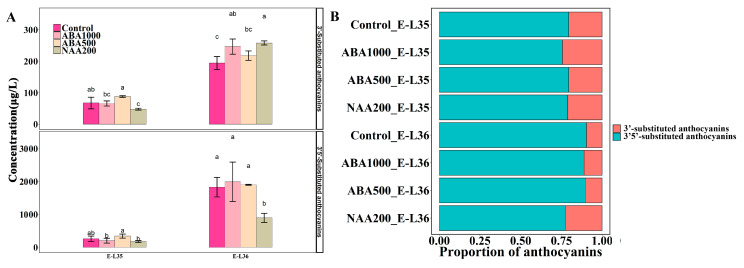
Effects of the treatments on the 3’-substituted anthocyanins and 3’5’-substituted anthocyanins: (**A**) changes in their concentrations and (**B**) proportions. Different letters indicate significant differences (*p* = 0.05).

An ANOVA analysis was to further highlight the impact of ABA and NAA on the accumulation of metabolites. A total of 15 anthocyanins were detected in the grapes, including nine 3′5′-substituted anthocyanins and six 3′-substituted anthocyanins. There were similar concentrations of anthocyanins in all samples at E-L 35, whereas the NAA200-treated berries contained lower levels compared with t other groups at E-L 36 (Figure 8). There were 14 norisoprenoids, seven terpenes, and 13 volatile C6/C9 compounds detected in this study. The total norisoprenoid content was much higher in the NAA200 than the controls at both the E-L 35 and E-L 36 stages and was unchanged with ABA. Regarding volatile C6/C9 compounds, their production was significantly increased in ABA500-treated and NAA200-treated berries at E-L 35 stage, but no significant difference was found at E-L 36 stage. The total terpenes were significantly increased in the ABA1000 berries at E-L 36 (Figure 8). At E-L 36, a higher level of the 3′-substituted anthocyanins was observed in the ABA1000, and NAA200 treated grape berries compared to the controls (Figure 9). NAA200 resulted in decreased 3′5′-substituted anthocyanins at E-L 36. Norisoprenoids, *β*-damascenone, and *β*-ionone, had low thresholds and pleasant smells [27,28], both (*Z*)-*β*-damascenone and (*E*)-*β*-damascenone were markedly increased in NAA200-treated berries at both stages (Figure S1). Some other norisoprenoids such as TPB, TDN, and TCH also had higher levels in NAA-treated berries. However, the concentrations of *β*-ionone, vitispirane A, vitispirane B, riesling acetal, trans-theaspirane, cis-theaspirane, and 6-methyl-5-hepten-2-one, *β*-cyclocitral were not significantly altered by the treatments. NAA treatment also had a positive effect on geranylacetone at E-L 35 (Figure S1). The concentrations of 4-terpineol were elevated by NAA200, and higher levels of linalool were found in ABA100 at E-L 36, while other terpenes were not affected (Figure S2). The concentrations of (*Z*)-3-hexenal, (*Z*)-2-hexenal, and (*E*)-2-hexenal were elevated in the ABA500 and NAA200 grapes at E-L 35 and ethyl hexanoate, cis-3-hexenyl acetate, and hexyl acetate were not altered by treatments. At E-L 36, (*E*)-3-hexen-1-ol and 1-hexanol were decreased in the ABA1000 (Figure S3). Overall, both terpenes and C6/C9 compounds showed an inconsistent response to the treatments.

### 2.5. Berry Transcriptome Analysis Supports the Metabolic Plasticity

**Figure 10:**
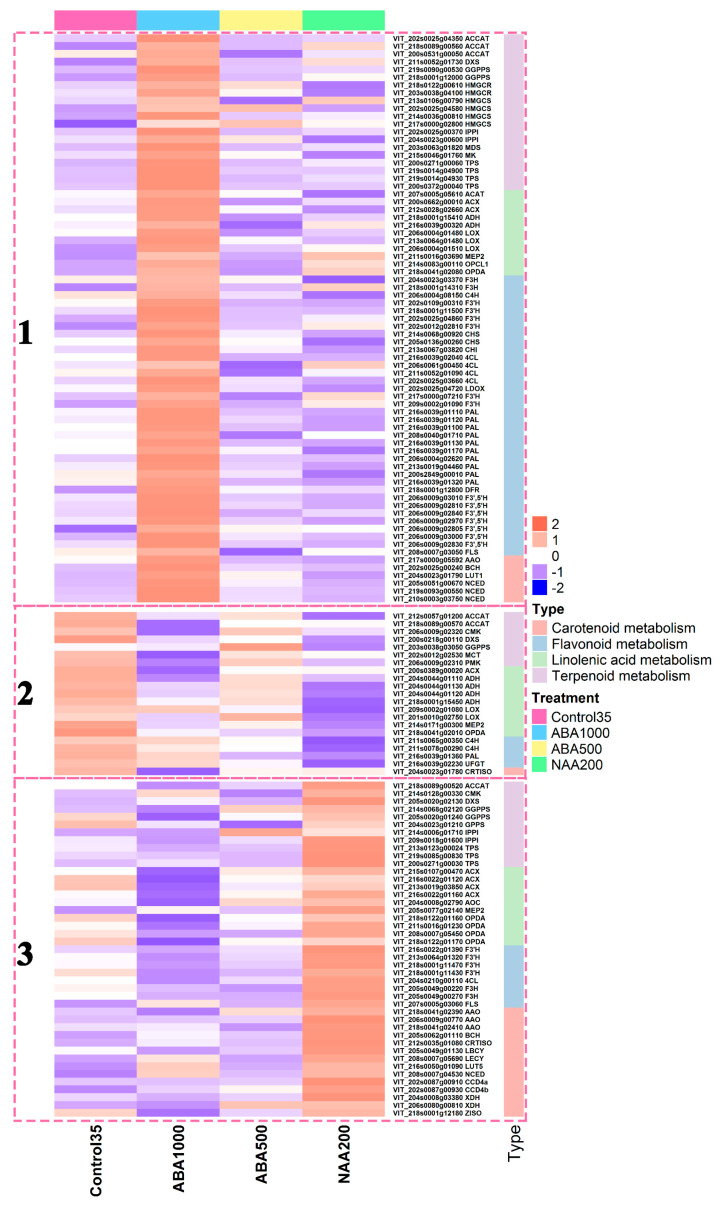
Heatmap analysis of gene involved in carotenoid, flavonoid, linolenic acid, and terpenoid metabolism and hierarchical clustering. Genes are grouped into three clusters by k-means cluster analysis.

In order to dissect the gene expression profiles corresponding to the metabolic variations, genes involved in related metabolisms were analyzed at E-L 35 (Figure 10). Low abundance genes were removed (RPKM < 1), there were 48, 38, 21, and 30 genes involved in flavonoid, terpenoid, carotenoid, and linolenic acid metabolisms, respectively (Figure S4). We found that both the genes upstream of anthocyanin biosynthetic pathways, such as *VviPAL*, *Vvi4CL*, *VviCHS*, and the downstream genes such as *VviF3′H*, *VviF3′5′H*, *VviDFR*, *VviLDOX*, and *VviFLS* were concentrated in Cluster 1 and highly up-regulated by ABA1000, compared with the control. In NAA200-treated berries, the genes of 1 *VviPAL*, 2 *VviC4H*, 1 *VviUFGT* in Cluster 2 were down-regulated, only the *VviF3*′H in cluster 3 was up-regulated. Highly expressed *VviF3′H* induced by NAA200 benefited the accumulation of 3′-substituted anthocyanins. Dihydromyricetin is a common substrate to produce myricetin and the 3′5′-substituted anthocyanins in grapes. We found, *VviFLS* in ABA1000 berries was significantly up-regulated to improve the conversion of dihydromyricetin into myricetin, which could, in turn, inhibit the production of the 3′5′-substituted anthocyanins. In the NAA200 grapes, the low concentration of anthocyanins should be related to the low abundance of *VviUFGT* transcripts. The majority of the genes involved in terpenoid metabolism showed high expression levels in ABA1000 and NAA200-treated berries, including the genes encoding 1-deoxyxylulose-5-phosphate synthase (DXS) and terpene synthases (TPS) in clusters 1 and 3. In the NAA200-treated berries, higher concentrations of norisoprenoids may be attributed to up-regulated genes related to carotenoid metabolisms, such as *VviXDH*, *VviAAO*, *VviBCH*, *VviCRTISO*, *VviLBCY*, *VviLECY*, *VviLUT5*, *VviZISO*, *VviCCD4a*, and *VviCCD4b*. Among these, carotenoid cleavage dioxygenase (CCD) encoded by *VviCCD4a* and *VviCCD4b* can cleave carotenoids to yield norisoprenoids [29], and other genes also required for carotenoid synthesis. In the ABA1000-treated berries, the up-regulation of 3 *VviNCED*, 1 *VviAAO*, 1 *VviBCH*, and 1 *VviLUT1* were found in cluster 1 (Figure 10). Some genes required for linolenic acid metabolism, including 1 *VviACAT*, 2 *VviACX*, 2 *VviADH*, 3 *VviLOX*, *VviMEP2*, 1 *VviOPCL1* and 1 *VviOPDA* were up-regulated in the ABA1000-treated berries in cluster 1. However, compared to the control, the other genes belonging to the families of *VviACX*, *VviADH*, *VviLOX*, *VviMEP2*, and *VviOPDA* were down-regulated in the groups of ABA1000 and NAA200 (cluster 2).

### 2.6. Validation by Quantitative Real-Time PCR

To validate the expression profiles obtained from the RNA-seq data, 10 genes associated with the biosynthesis of the main sensory compounds were selected for qRT-PCR analysis. There were five genes belonging to the phenylpropanoid/flavonoid biosynthetic pathway (*VviPAL*, *VviF3H*, *VviF3’5’H*, *VviLAR*, and *VviAOMT*), two genes (*VviNCED3* and *VviCCD4a*) involved in the synthesis of ABA and norisoprenoids, and three genes (*VviSUS*, *VviSUC27*, and *VviPL*) relating to the regulation of synthesis and transport of sucrose and berry softening (Figure S5). The internal controls were *VviUbiquitin1* and *Vviβ-Actin*. The results showed that the expression levels of 10 genes determined by qRT-PCR were significantly correlated with those from the RNA-seq data at the 0.01 level (r = 0.83), which verified the reliability of the RNA-seq analysis.

## 3. Discussion

In this study, we investigated the effects of ABA and NAA on Cabernet Sauvignon grape berries based on E-L stage with similar sugar levels, the purpose of which is to eliminate the possible influence of sugar or other developmental characteristics. Sugar can enhance anthocyanin accumulation and related gene expression in grape berries [30] and may impact the biosynthesis of other flavor compounds. We observed that the application of 500 mg/L ABA had insignificant effects on sugar increase and acid decline, and the ripening process of berries was similar to the control, suggesting that only very small amounts of exogenous ABA could penetrate into the berry due to the wax barrier on the peel and the rapid solution evaporation, and the amount may be not enough to modify the berry maturation progression. However, there are still a few differences in the concentration of individual compounds and the expression of some genes between ABA500 and the control groups (Figure 3 and Figure 6). Similarly, Ferrara et al. observed that 400 mg/L ABA application has no effect on total soluble solids, pH, titratable acidity but can increase the anthocyanin concentration of the berry skin [17].

The proportion of cyanidin-derived (3′-substituted) anthocyanin metabolites was significantly increased in both ABA1000 and NAA200-treated grape berries (Figure 9B). It appeared that the F3′H branch, in contrast with the F3′5′H branch, in the flavonoid metabolism, was more sensitive to exogenous ABA and NAA stimulation. The influence of the ABA1000 and NAA200 on the accumulation of anthocyanins in grape berries has been previously studied [13,14,17,31], but scarce attention was paid to the two branch pathways driven by F3′H and F3′5′H. The final metabolites from these two pathway branches, 3′-substituted and 3′5′-substituted anthocyanins possess different color expressions, and they jointly determine the overall color appearance of red grape berries and corresponding wine [32]. The present results indicate that the skin color of grapes could shift from the purple-red to the red hue when the natural ripening speeds of grape berries are changed by exogenous ABA and NAA. Another important finding was that exogenous NAA markedly promoted the accumulation of norisoprenoids, with (*Z*)-*β*-damascenone and (*E*)-*β*-damascenone representing the major norisoprenoids. Although *β*-damascenone was found to be significantly higher in wine made with NAA-treated berries [18], the reason has not been determined. In this study, we found that the NAA application markedly up-regulated the expression of *VviCCD4a* and *VviCCD4b* in cluster 3 (Figure 10), which are tightly associated with *β*-damascenone and other C13-norisoprenoids accumulation [29]. The upstream genes related to carotenoid and norisoprenoid biosynthesis such as *VviDXS*, *VviGGPPS*, *VviLBCY*, *VviLECY,* and *VviZISO* were also found to show a higher level in NAA-treated berries (Figure 10). The up-regulated DEGs identified at E-L 35 in NAA-treated berries compared to Control35 were enriched in “photosynthesis” pathways (Table S4). The carotenoids, as the precursors of norisoprenoids, act as light-harvesting antenna pigments and play important roles in the photoprotection of the plant [33]. It indicated that the NAA application might have a positive effect on photosynthesis and concurrently increase carotenoid levels. Therefore, the higher concentrations of norisoprenoids in NAA-treated berries could result from elevated substrate availability combined with the up-regulation of key genes (*VviCCD4a* and *VviCCD4b*). Although the concentrations of these quality-related compounds are shown to highly correlate with the expression of corresponding genes, this only partially explains that the increased compound concentration could result from the up-regulated expression of biosynthesis-related genes, because the levels of the compounds actually depend on both their synthesis and degradation. It is also important to assess the expression of genes in the degradation of metabolism. However, the knowledge about the genes involved in the degradation of anthocyanins and norisoprenoids is still very limited in grape berry, which needs more research to dissect the balance between the production and degradation of the flavor compounds induced by ABA and NAA in grape berry. Moreover, although gene expression analyses have offered insights into the mechanism underlying the responses of grape berries to ABA and NAA treatments, other regulations such as alternative splicing, protein levels, and enzyme activity also play roles in controlling the accumulation of metabolites. Therefore, a multi-omics integrated analysis in the future study, including transcriptome, proteome, and others, can help us understand the influences of ABA and NAA on quality-related metabolites in grape berry.

The genes responsible for varied growth-to-ripening transitions were found by transcriptomic analysis (Table S1). The common genes in Figure 3B should be physiologically involved in berry developmental transitions from E-L 33 to E-L 35, and the genes in the sets of C_only, A1_only, A2_only and N_only were speculated to determine the growth-to-ripening transition rate. Some of these genes have been reported to relate to ripening events like berry softening and sugar metabolism, but the roles of other genes that may trigger ripening were still uncovered. These DEG sets can be functionally verified in the future. Genes that were regulated by both ABA1000 and NAA200 at E-L 35 were identified (Table S5), for instance, 1 *VviPG* was up-regulated by ABA1000 while down-regulated by NAA200 and genes encoding ABA signaling protein PP2C, auxin-responsive protein, and auxin-induced protein were down-regulated by ABA1000 while up-regulated by NAA200. These genes seem to play vital roles in hormonal control and contribute to the various ripening timings. The different responses of the above-mentioned ABA and auxin signaling genes indicate that there is tight cross-talk between ABA and auxin in grape berry.

In this study, the authors only focused on the effects of ABA and NAA on the early ripening process of grape berries due to the importance of véraison during berry development. However, the long-term effects of the two plant growth regulators on grape berries in combined transcriptomic and metabolic levels remain unclear, especially the volatile compounds when berries arrived at maturity (E-L 38). It has been reported that pre-véraison ABA has a long-term effect (beyond 40 days after véraison) on grape berries on the basis of the regulation of phenylpropanoid-related gene expression and metabolite content [31]. The deeper and more complicated relationship between hormones and flavor compounds should be considered and studied further. Climate change has a profound impact on grape berry ripening [34], improved understanding in relation to the control of ripening activation and duration is vital for viticulturists and winemakers to successfully cope with the changing climate and harvest grape berries with desirable flavors.

## 4. Materials and Methods

### 4.1. ABA and NAA Application in Vineyard and Grape Berry Sampling

ABA and NAA application was performed in a commercial six-year-old *Vitis vinifera* L. cv. Cabernet Sauvignon vineyard with yellow earth and gravel loam located in the Chateau Changyu Afip Global (44°30′ N, 116°80′ E) of Miyun county, Beijing, China in 2014. ABA and NAA were dissolved in an aqueous solution with 0.05% (v/v) Tween 20 to yield 1000 mg/L ABA, 500 mg/L ABA and 200 mg/L NAA solutions, named as ABA1000, ABA500, and NAA200, respectively. The 0.05% (v/v) Tween 20 solution was used as the control. The solutions were sprayed directly onto the grape berries before the véraison stage (at E-L 33) when the TSS of the berries was about 4 °Brix (Figure 1). To avoid rapid evaporation of the solution, the application was carried out at sunset. The second spray application was performed after the first spray solution was fully absorbed. A random block design was used, and each treatment had three biological replicates with about 50 vines in each replicate. The berries were harvested according to a modified E-L classification system published in a previous study [35]. At E-L 33: berries still hard and green, E-L 35: the onset of coloration and softening and E-L 36: berries with intermediate Brix values (around 14 °Brix), about 500 grape berries from each replicate were randomly collected and immediately transported to our laboratory. Afterward, the berries were carefully washed with distilled water to remove the residues on the surface. Approximately 100 berries were used for the physicochemical index analysis, and the rest were frozen using liquid nitrogen and stored at −80 °C for further analysis.

### 4.2. Physicochemical Index Analysis

A subsample of 100 berries was weighed and pressed by a hand juice crusher. The TSS (°Brix) of the juice was measured using a digital handheld pocket Brix refractometer (PAL-2, ATAGO, Tokyo, Japan). After the juice was diluted five-fold using deionized water, the titratable acidity (expressed as g tartaric acid equivalents per liter of juice) was determined by adjusting the pH of the grape juice to 8.2 using NaOH [36].

### 4.3. RNA Extraction and Sequencing

The berry samples used for RNA extraction and transcriptome sequencing included the control grape berries harvested at E-L 33 stage (Control33) and all the samples harvested at E-L 35 stage (ABA1000, ABA500, NAA200, and Control35). Approximately 50 berries were randomly selected from each biological replicate for the total RNA extraction. Before extraction, all seeds were manually removed. The extraction followed a plant RNA isolation kit according to the manufacture’s protocol (Sigma RT-250, St. Louis, MO, USA). The total RNA content quality and quantitation were assessed using a Qubit 2.0 fluorometer RNA Assay Kit (Life Technologies, Carlsbad, CA, USA) and Agilent 2100 Bioanalyzer (Agilent, Santa Clara, CA, USA). A total of 16 RNA-seq libraries were constructed (4 × Control33, 3 × Control35, and 3 × each treatment at stage of E-L 35). The RNA sequencing was performed using Illumina HiseqTM2000 (Illumina Inc., San Diego, CA, USA) to yield 50-bp single-end reads. After filtering out the low-quality reads, a total of 396 million clean reads were obtained, then mapped to the grape reference genome using TopHat and annotated in comparison with the V2.1 version (http://genomes.cribi.unipd.it/grape/). The mapping rate of the clean reads all exceeded 80% for the respective RNA-seq libraries, which indicated that the sequencing quality was sufficient for further data mining. The expression levels of genes were normalized via calculating the target Reads Per Kilobases Per Million Reads (RPKM) value. An R package (DESeq2) was used to analyze the differentially expressed genes (DEGs), and the significance of the DEGs was judged based on the False Discovery Rate ≤ 0.01 and the absolute value of log2Ratio ≥ 1. Additionally, information from the Clusters of Orthologous Groups of proteins (COGs), Kyoto Encyclopedia of Genes and Genomes (KEGG), Gene Ontology (GO), and NCBI non-redundant protein sequences (Nr) database were annotated to all genes.

### 4.4. Quantitative Real-Time PCR

Quantitative real-time PCR (qRT-PCR) was conducted to verify the RNA-Seq data. Total RNA was isolated using the same method mentioned above. Then, the total RNA was reverse-transcribed into cDNA using 5 x TransScript^®^ All-in-One-Step SuperMix for qPCR and gDNA Remover (TransGen, Beijing, China) following the manufacturer’s instructions. Quantitation was performed using SYBR^®^ Premix Ex TaqTM (Takara, Otsu, Japan) on a 7300 Real-Time PCR System (Applied Biosystems, Foster City, CA, USA). qRT-PCR was performed on three independent biological replicates, with each replicate containing three technical replicates. Each real-time PCR reaction (20 μL) contained 2.0 μL of the cDNA template, 1.6 μL of the primer mixture (10 μM each, mixed with equivalent volume), 10.0 μL of 2×SYBR Premix Ex Taq II and 0.4 L of 50× ROX Reference Dye (Takara, Otsu, Japan), and 6.0 μL ddH2O. Reactions were performed via a two-step method: 95 °C for 30 s, 40 cycles of 95 °C for 10 s, 60 °C for 31 s, and a melt cycle from 60 °C to 95 °C. Primer information is available from Table S6 [32,36,37,38,39,40,41,42,43,44,45,46]. The normalized relative expression levels of target genes were calculated by 2-∆Ct (∆Ct = CtTarget - CtRefGene, Ct: cycle threshold) using *VviUbiquitin1* and *Vviβ-Actin* as reference genes. CtRefGene represented the geometric mean of Ct values of the two reference genes. The target genes, *VviUbiquitin1* and *Vviβ-Actin*, were analyzed simultaneously.

### 4.5. Determination of Anthocyanins

Anthocyanin extraction was conducted previously described [47]. Briefly, the freeze-dried grape skin powder (0.5 g) was mixed with 10 mL of formic acid: methanol (2:98, v/v). The mixture was sonicated for 10 min and then shaken at 150 rpm in the darkness for 30 min at 25 °C. Then the mixture was centrifuged at 8000× *g* for 10 min to collect the supernatant. The extraction procedure was repeated four times, and all the supernatant portions were pooled. Subsequently, the pooled supernatant was evaporated at 28 °C in a rotary evaporator (Shanghai Yarong Biochemical Instrument Factory, Shanghai, China) to remove methanol. The dryness was re-dissolved in 10 mL of a solution consisting of 90% mobile phase A (0.01% v/v formic acid in water): 10% mobile phase B (0.1% v/v formic acid in 50:50 v/v acetonitrile: methanol). The re-dissolved extract was filtered through a 0.45-µm polyether sulfone membrane prior to HPLC-MS analysis. An Agilent series 1200 HPLC coupled with an Agilent 6410 QqQ mass spectrometer (Agilent Technologies, Santa Clara, CA, USA) was used for the identification and quantification of the anthocyanins in the grape berries according to our previous report [48]. A Poroshell 120 EC-C18 column (150 × 2.1 mm, 2.7 µm, Agilent Technologies, Santa Clara, CA, USA) was used. Multiple reactions monitoring (MRM) mode was selected for both identification and quantification. Each biological replicate was conducted in duplicate.

### 4.6. Volatile Compounds Determination

One hundred grape berries, after removing the seeds, were blended with 1 g PVPP, and ground into powder in liquid nitrogen. The flesh was macerated at 4 °C for 4 h, and immediately centrifuged at 8000 rpm for 15 min to obtain the clear juice. Headspace solid-phase micro-extraction (HS-SPME) of volatile compounds from the grape samples followed a published method [49]. Glycosidic precursors were extracted by adsorption on Cleanert PEP-SPE cartridge (150 mg/6 mL; Bonna-Agela Technologies, Beijing, China). Before 2 mL of clear juice was added, the PEP-SEP cartridge was preconditioned with 10 mL methanol and 10 mL water separately. Then the cartridge was washed with 2 mL water and 5 mL dichloromethane to remove sugars, and polar compounds, and most free volatiles were eluted with 20 mL methanol to obtain the aroma precursors. The methanol extract was evaporated to dryness under a nitrogen stream and re-dissolved in 10 mL of citrate-phosphate buffer solution (0.2 M, pH = 5.0) and added to 100 µL AR2000 (Rapidase, 100 g/L). Enzymatic hydrolysis was conducted in an incubator at 40 °C for 16 h. The released volatiles were extracted with HS-SPME. An Agilent 6890 gas chromatography coupled with an Agilent 5975C mass spectrometer were used to analyze the volatile compounds in the samples. An HP-INNOWAX capillary column (60 m × 0.25 mm × 0.25 μm, J&W Scientific, Folsom, CA) were used. Volatile compounds in the samples were identified by comparing their retention time and mass spectrums with the available external standards. The volatiles, without reference standards were tentatively identified by comparing their retention indices and mass spectrums with the NIST11 database. Quantitation of these volatile compounds followed our previously published method [49]. A synthetic matrix containing 200 g/L glucose and 7 g/L tartaric acid was adjusted to pH 3.3. The external standards were dissolved to the synthetic matrix in fifteen successive concentrations. The synthetic matrix was extracted using the same headspace solid-phase micro-extraction method and analyzed based on the same GC-MS conditions. The volatile compounds with available standards were quantified using their reference standards, whereas the volatiles without available standards were quantified using standards that had the same functional groups and similar numbers of carbon atoms.

### 4.7. Statistical Analyses

Data were expressed as the mean ± standard deviation of triplicate tests. One-way analysis of variances (ANOVA) was carried out to compare the differences among the means under Turkey multiple range test at a significant level of 0.05 using SPSS Statistical 21.0 (SPSS Inc., Chicago, IL, USA). OPLS-DA was conducted using SIMCA (version 14.1 from Umetrics, Umea, Sweden). A hierarchical cluster analysis of metabolites was performed using the Expander R package “ComplexHeatmap.”

## Supplementary Materials

Supplementary materials can be found at https://www.mdpi.com/2223-7747/9/5/630/s1. Figure S1: Change in the concentration of 14 norisoprenoids in different treatments; Figure. S2: Change in the concentration of eight terpenes in different treatments; Figure. S3: Change in the concentration of seven C6/C9 compounds in different treatments; Figure S4: Metabolisms of terpenoid, carotenoid, linolenic acid, and flavonoid. Abbreviations: ACCAT, acetyl-CoA C-acetyltransferase; HMGCS, hydroxymethylglutaryl-CoA synthase; HMGCR, hydroxymethylglutaryl-CoA reductase; MK, mevalonate kinase; PMK, phosphomevalonate kinase; PPMD, diphosphomevalonate decarboxylase; DXS, 1-deoxy-D-xylulose-5-phosphate synthase; DXR, 1-deoxy-D-xylulose-5-phosphate reductoisomerase; MCT, 2-C-methyl-D-erythritol 4-phosphate cytidylyltransferase; CMK, 4-diphosphocytidyl-2-C-methyl-D-erythritol kinase; MDS, 2-C-methyl-D-erythritol 2;4-cyclodiphosphate synthase; HDS, E-4-hydroxy-3-methylbut-2-enyl-diphosphate synthase; HDR, 4-hydroxy-3-methylbut-2-en-1-yl diphosphate reductase; IPPI, isopentenyl-diphosphate Delta-isomerase; GPPS, geranyl diphosphate synthase; FPPS, farnesyl diphosphate synthase; GGPPS, geranylgeranyl diphosphate synthase; PSY, phytoene synthase; PDS, 15-cis-phytoene desaturase; ZDS, zeta-carotene desaturase; ZISO, zeta-carotene isomerase; CRTISO, prolycopene isomerase; LECY, lycopene epsilon-cyclase; LBCY, lycopene beta-cyclase; LUT5, beta-ring hydroxylase; BCH, beta-carotene 3-hydroxylase; LUT1, carotene epsilon-monooxygenase; ZEP, zeaxanthin epoxidase; VDE, violaxanthin de-epoxidase; NCED, 9-cis-epoxycarotenoid dioxygenase; XDH, xanthoxin dehydrogenase; NSY, neoxanthin synthase; AAO, abscisic-aldehyde oxidase; CCD, carotenoid cleavage dioxygenase; LOX, lipoxygenase; HPL, hydroperoxide lyase; ADH, alcohol dehydrogenase; CHAT, Z-3-hexen-1-ol acetyltransferase; AOS, allene oxide synthase; Aoc, allene oxide cyclase; OPDA, 12-oxophytodienoic acid reductase; OPCL1, OPC-8:0 CoA ligase 1; ACX, acyl-CoA oxidase; MEP2, glyoxysomal fatty acid beta-oxidation multifunctional protein MFP-a; ACAT, acetyl-CoA acyltransferase; JOMT, jasmonate O-methyltransferase; PAL, phenylalanine ammonia-lyase; C4H, cinnamate 4-hydroxylase; 4CL, 4-coumarate: CoA ligase; CHS, chalcone synthase; CHI, chalcone isomerase; F3’H, flavonoid 3’-hydroxylase; F3’5’H, flavonoid 3’,5’-hydroxylase; F3H, flavanone 3-hydroxylase; FLS, flavonol synthase; DFR, dihydroflavonol reductase; LDOX, leucoanthocyanidin dioxygenase; UFGT, UDP-glucose: flavonoid 3-O-glucosyltransferase; AOMT, anthocyanin o-methyltransferase; AAT, anthocyanin acyltransferase; Figure S5: Validation of RNA-seq results by quantitative real-time PCR. The left-hand heatmap indicates the average value of the relative expression from three independent real-time PCR experiments. The right-hand heatmap indicates the average FPKM values of the RNA-seq analysis. Correlation is significant at the 0.01 level; Table S1: Differentially expressed gene (DEG) sets responsible for growth-to-ripening transition; Table S2: List of the variables with VIP value larger than 1; Table S3: List of compounds corresponding to the symbols in loading plot of Figure 4; Table S4: KEGG enrichment analysis of Differentially expressed genes (DEGs) at E-L 35 stage; Table S5: List of the genes regulated by both ABA1000 and NAA200 at E-L 35 stage; Table S6: List of the primers used for quantitative real-time RT-PCR validation experiments.

## Abbreviations

ABA	Abscisic acid
NAA	α-Naphthaleneacetic acid
SA	Salicylic acid
CL	Changli
GT	Gaotai
IAA	Indole-3-acetic acid
BTOA	Benzothiazole-2-oxyacetic acid
DEG	Differentially expressed genes
PE	Pectinesterase
PG	Polygalacturonase
PL	Pectate lyase
NCED	9-Cis-epoxycarotenoid dioxygenase
SUC	Sucrose transporter
SUS	Sucrose synthase
MDH	Malate dehydrogenase
PYR/PYL	Pyrabactin resistance/pyrabactin-like
PP2C	Type 2C protein phosphates
TGA	TGACG motif-binding factor
BRI1	Brassinosteroid-insensitive1
BR	Brassinosteroid
BAK1	BRI1 associated receptor Kinase 1
AUX1	Auxin influx carrier protein
AUX/IAA	Auxin-responsive proteins
TCH4	Xyloglucan:xyloglucosyl transferase
F3′H	Flavonoid-3’-hydroxylases
UFGT	UDP glucose flavonoid-3-O-glucosyltransferase
PSY	Phytoene synthase
CCD	Carotenoid cleavage dioxygenase
RPKM	Reads per kilobases per million reads
FDR	False discovery rate
KEGG	Kyoto encyclopedia of genes and genomes
GO	Gene ontology
Nr	NCBI non-redundant protein sequences
qRT-PCR	Quantitative real-time PCR
