# Supplementary material for: Variation of Growth-to-Ripening Time Interval Induced by Abscisic Acid and Synthetic Auxin affecting Transcriptome and Flavor Compounds in Cabernet Sauvignon Grape Berry
Source: Plants (Basel). 2020 May 14;9(5):630. doi: 10.3390/plants9050630 (PMC7286024; doi:10.3390/plants9050630)
Supplement: Supplementary file 1 [file plants-09-00630-s001.zip › Supplementary file/Supplementary figure.docx]

Supplementary figures:


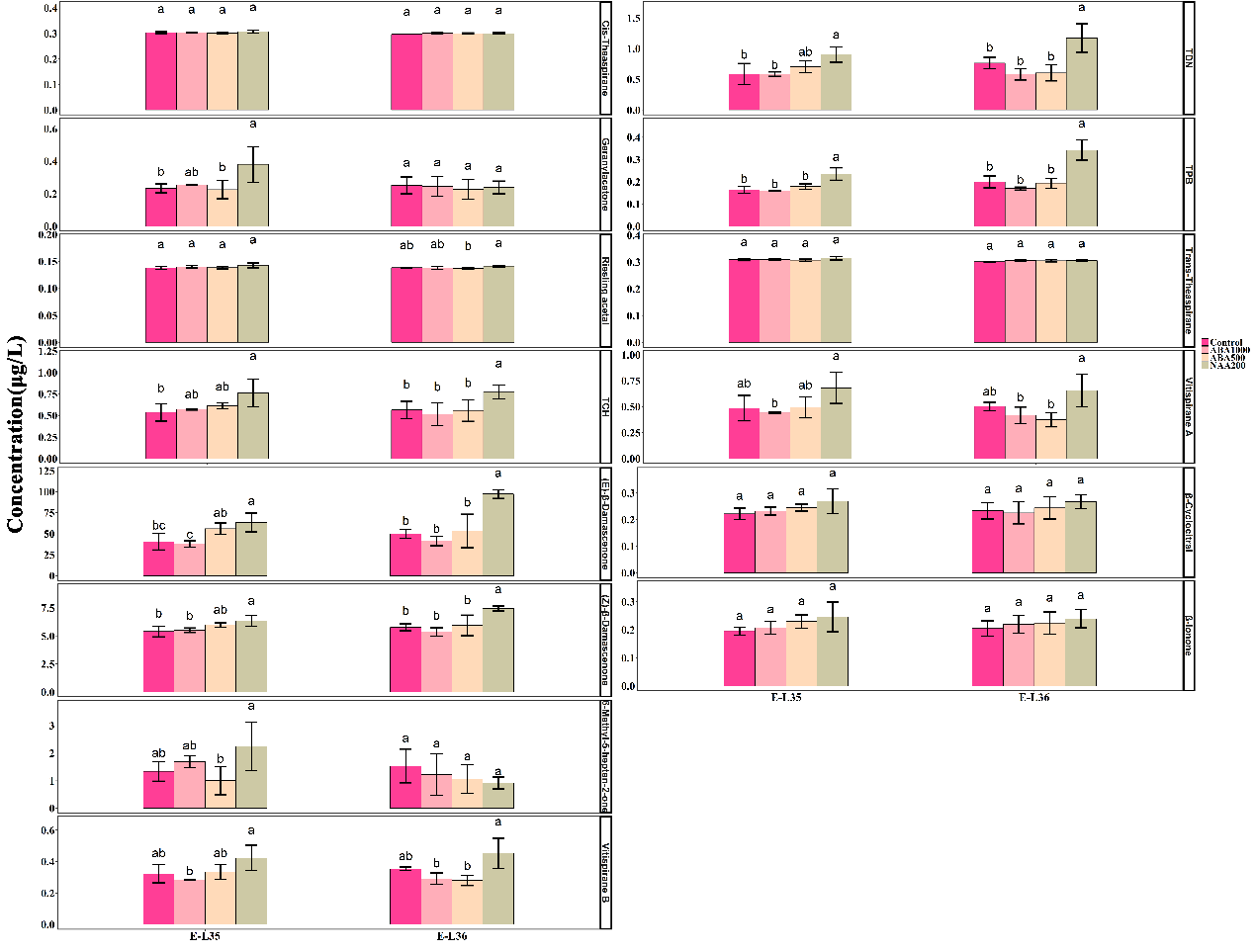


**Figure S1:** Change in the concentration of 14 norisoprenoids in different treatments.


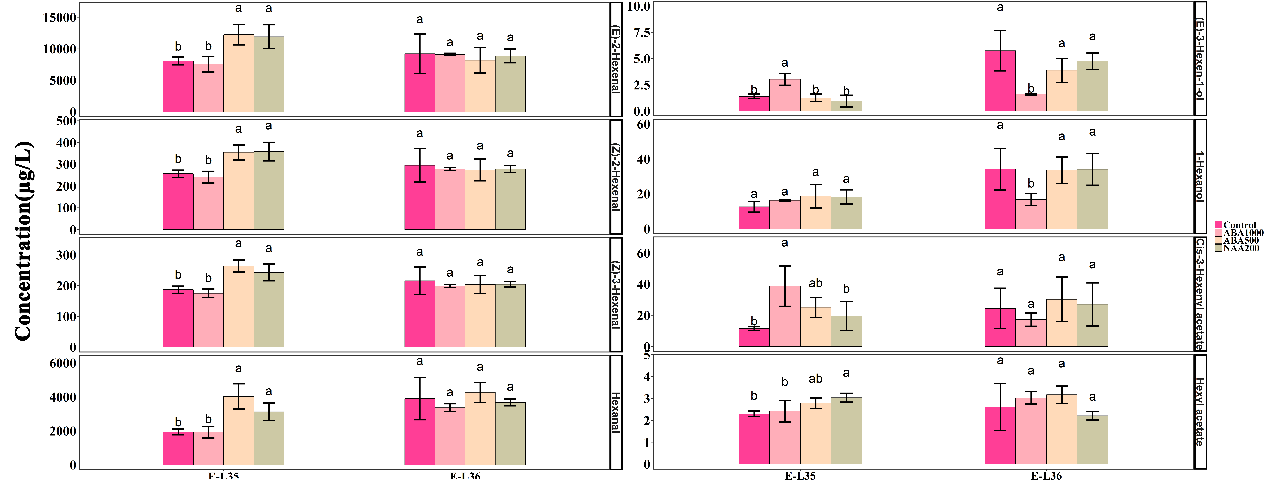


**Figure S2:** Change in the concentration of 8 terpenes in different treatments.


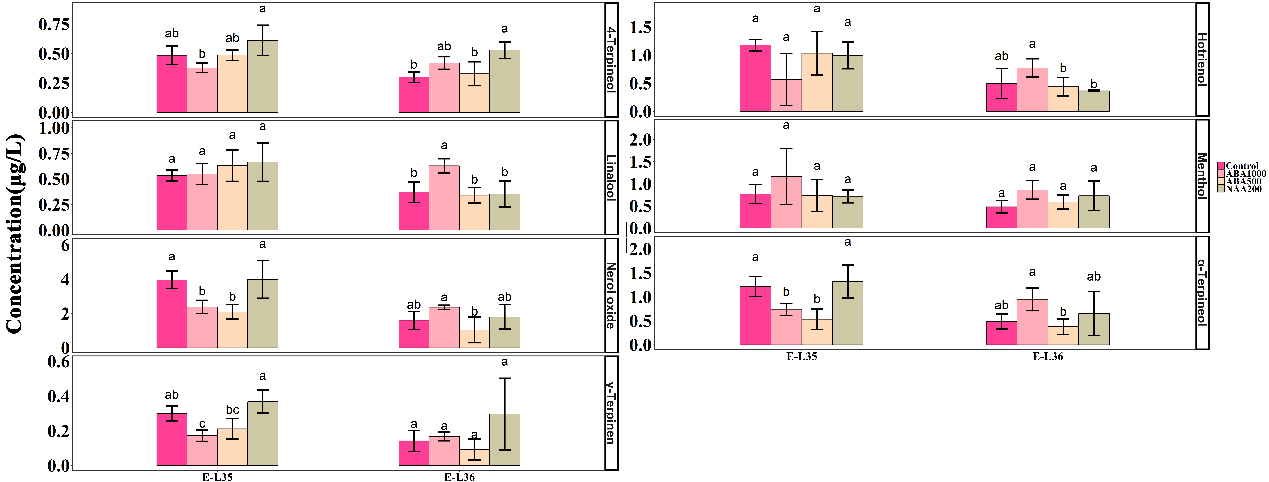


**Figure S3:** Change in the concentration of 7 C6/C9 compounds in different treatments.


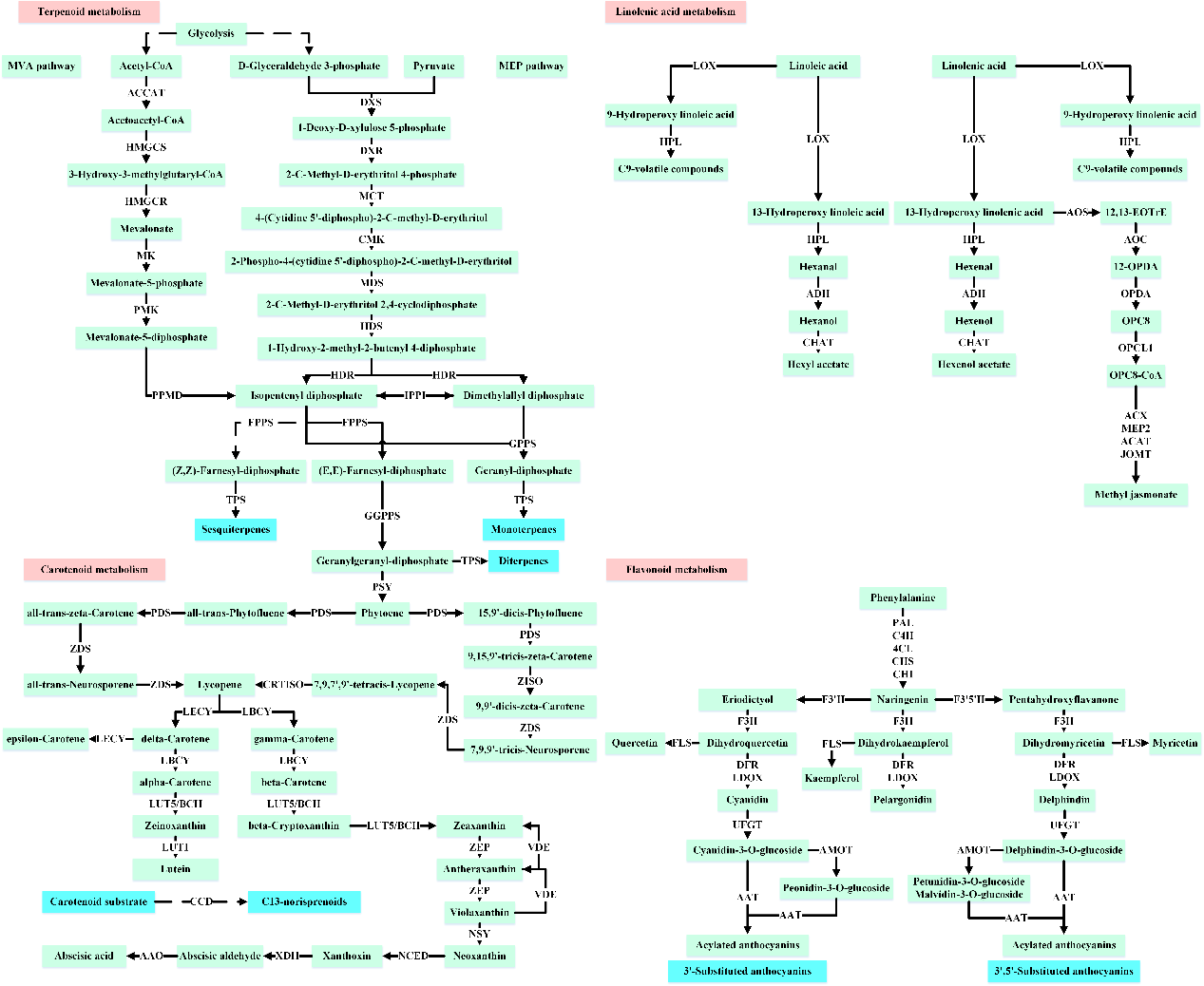


**Figure S4:** Metabolisms of terpenoid, carotenoid, linolenic acid and flavonoid. Abbreviations: ACCAT, acetyl-CoA C-acetyltransferase; HMGCS, hydroxymethylglutaryl-CoA synthase; HMGCR, hydroxymethylglutaryl-CoA reductase; MK, mevalonate kinase; PMK, phosphomevalonate kinase; PPMD, diphosphomevalonate decarboxylase; DXS, 1-deoxy-D-xylulose-5-phosphate synthase; DXR, 1-deoxy-D-xylulose-5-phosphate reductoisomerase; MCT, 2-C-methyl-D-erythritol 4-phosphate cytidylyltransferase; CMK, 4-diphosphocytidyl-2-C-methyl-D-erythritol kinase; MDS, 2-C-methyl-D-erythritol 2;4-cyclodiphosphate synthase; HDS, E-4-hydroxy-3-methylbut-2-enyl-diphosphate synthase; HDR, 4-hydroxy-3-methylbut-2-en-1-yl diphosphate reductase; IPPI, isopentenyl-diphosphate Delta-isomerase; GPPS, geranyl diphosphate synthase; FPPS, farnesyl diphosphate synthase; GGPPS, geranylgeranyl diphosphate synthase; PSY,phytoene synthase; PDS, 15-cis-phytoene desaturase; ZDS, zeta-carotene desaturase; ZISO, zeta-carotene isomerase; CRTISO, prolycopene isomerase; LECY, lycopene epsilon-cyclase; LBCY, lycopene beta-cyclase; LUT5, beta-ring hydroxylase; BCH, beta-carotene 3-hydroxylase; LUT1, carotene epsilon-monooxygenase; ZEP, zeaxanthin epoxidase; VDE, violaxanthin de-epoxidase; NCED, 9-cis-epoxycarotenoid dioxygenase; XDH, xanthoxin dehydrogenase; NSY, neoxanthin synthase; AAO, abscisic-aldehyde oxidase; CCD, carotenoid cleavage dioxygenase; LOX, lipoxygenase; HPL, hydroperoxide lyase ;ADH, alcohol dehydrogenase; CHAT, Z-3-hexen-1-ol acetyltransferase; AOS, allene oxide synthase; Aoc, allene oxide cyclase; OPDA, 12-oxophytodienoic acid reductase; OPCL1, OPC-8:0 CoA ligase 1; ACX, acyl-CoA oxidase; MEP2, glyoxysomal fatty acid beta-oxidation multifunctional protein MFP-a; ACAT, acetyl-CoA acyltransferase; JOMT, jasmonate O-methyltransferase; PAL, phenylalanine ammonia-lyase; C4H, cinnamate 4-hydroxylase; 4CL, 4-coumarate: CoA ligase; CHS, chalcone synthase; CHI, chalcone isomerase; F3'H, flavonoid 3'-hydroxylase; F3'5'H, flavonoid 3',5'-hydroxylase; F3H, flavanone 3-hydroxylase; FLS, flavonol synthase; DFR, dihydroflavonol reductase; LDOX, leucoanthocyanidin dioxygenase; UFGT, UDP-glucose: flavonoid 3-O-glucosyltransferase; AOMT, anthocyanin o-methyltransferase; AAT, anthocyanin acyltransferase;


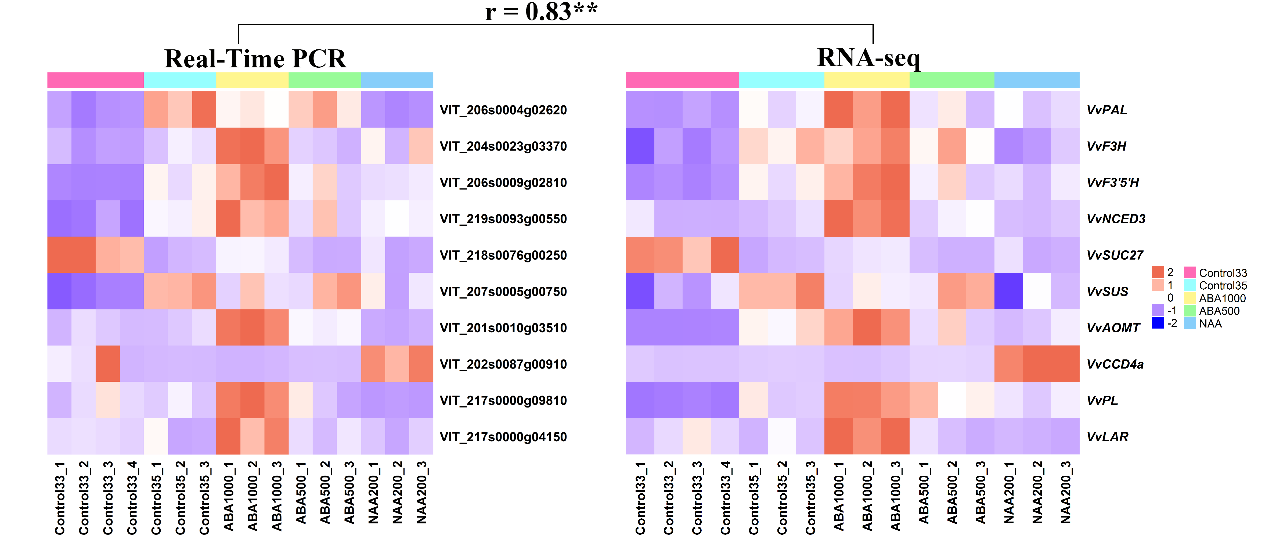


**Figure S5：**Validation of RNA-seq results by quantitative real-time PCR. The left-hand heatmap indicates the average value of relative expression from three independent real-time PCR experiments. The right-hand heatmap indicates the average FPKM values of the RNA-seq analysis. Correlation is significant at the 0.01 level.
